# Linking carbonatites, rare earth ores, and subduction-fertilized mantle lithosphere

**DOI:** 10.1126/sciadv.aeb2942

**Published:** 2026-04-08

**Authors:** Carl Spandler, Andrew S. Merdith, Amber Griffin

**Affiliations:** ^1^ Department of Earth Science, Adelaide University, Adelaide, South Australia.; ^2^ ARC Centre in Critical Resources for the Future, Perth, Australia.

## Abstract

Continental intraplate magmatism through time has played a crucial role in ancient mass extinctions, landscape evolution, global climate, and volcanism. These magmatic systems are also crucial sources of critical metals, including the rare earth elements (REEs). Nonetheless, the origins of alkaline intraplate magmas, such as carbonatites, remain enigmatic. Here, we use kinematic plate modeling to show that ~35% of the present-day subcontinental lithospheric mantle has experienced substantial subduction-related fertilization in the past 2 billion years. These fertilized mantle domains underlie ~67% of post–1.8-billion-year-old carbonatites and ~72% of magma-related REE ore deposits (and ~92% of Precambrian REE deposits), substantiating a genetic link between the ancient, enriched mantle lithosphere and alkaline intraplate magmatism and associated ore deposits. We find no correlation between the timing of mantle source fertilization and carbonatite formation, indicating that the evolution of these magmas is multistage, including an initial metasomatic “primer” of the mantle lithosphere and a subsequent and disconnected melting “trigger” event responsible for magma generation.

## Teaser

Continental intraplate magmatism and rare earth ores are linked to the ancient subduction-enriched mantle lithosphere.

## INTRODUCTION

Plate tectonics provides a robust and well-established framework to understand the origins of plate-margin magmatism in the present day and back through geologic time. More enigmatic is continental intraplate magmatism, despite occurring throughout Earth history and across all continents (*1*, *2*). Although experimental research has revealed the physical and chemical conditions needed to generate intraplate alkaline magmas via mantle melting (*3*–*6*) and a range of geologic triggers for magma production have been proposed (*6*–*9*), a holistic understanding of these magma systems remains elusive. Resolving the origins of intraplate magmatism is crucial for understanding magmatic processes on Earth and is of importance to defining locations of metal resources to sustain future society as these magmatic rocks [such as carbonatites; (*6*)] are major sources of critical metals, including rare earth elements (REEs) (*10*, *11*).

**Fig. 1.**
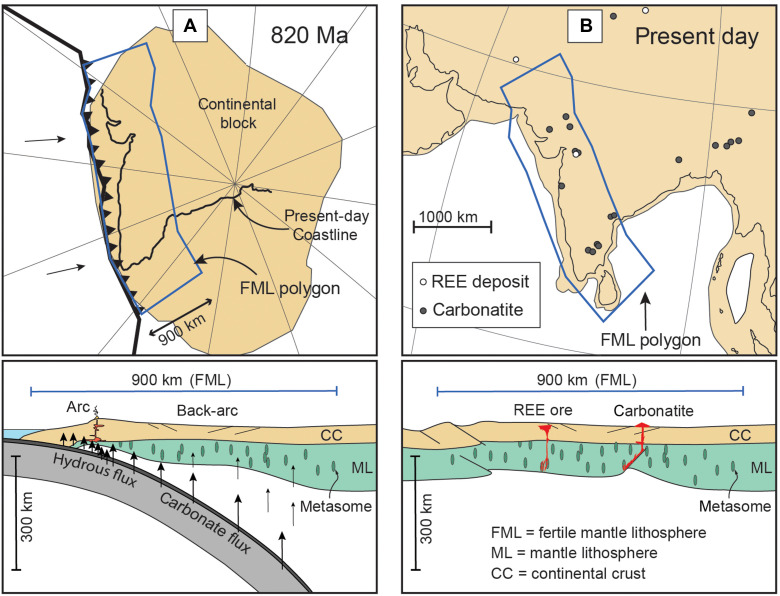
Schematic illustration of the model parameters of this study, using the Indian continent block as an example. (**A**) Reconstructed Neoproterozoic continental convergent plate margin (from GPlates), together with the present-day coastline of India for reference. The cross section depicts the 900-km-wide zone of mantle lithosphere metasomatized by subducting slab-derived fluids or melts. This zone is represented by the blue FML polygon in the plate reconstruction. (**B**) Same continental block and associated FML polygon in the present day, with the location of carbonatites and REE deposits (i.e., formed from anorogenic processes). Note that, for simplicity to demonstrate our approach, the figure only displays a single polygon. Ma, million years.

Intraplate magma occurrences have been linked to the arrival of mantle plumes at the base of the lithosphere (*7*, *12*, *13*). However, many carbonatites present no evidence links to plumes (*14*), and plumes are generally considered to be relatively hot and therefore are difficult to attribute as the source of low-temperature alkaline magmas, such as carbonatites (*2*, *3*). Instead, models implicating plate tectonics have been gaining attention. In these models alkaline magmatism (e.g., in a continental rift or intraplate extension zone) forms via melting of fertilized mantle lithosphere (FML) that was metasomatized via a stage of plate subduction that predates the magmatism (*2*, *4*, *14*–*16*) (Fig. 1). During subduction, a fluid flux of volatiles (H_2_O, CO_3_^2−^, and halogens) and incompatible elements is expected to migrate from the subducting slab into the overriding mantle lithosphere inboard of the convergent margin (Fig. 1). This slab flux is widely accepted to be implicit in the generation of active arc magmatism (*17*) but may also react with mantle peridotite to produce metasomatic domains or “metasomes” (*4*, *5*, *18*) within the mantle lithosphere of the overriding plate that can survive long after cessation of plate convergence. Subsequent melting of these metasomes is postulated to explain a variety of intraplate alkaline magma types (*4*, *5*, *18*, *19*). Of relevance to carbonatites are recent works demonstrating that subducted carbon (via carbonate-bearing oceanic crust, sediments, and ophicarbonate) can be efficiently recycled from the slab at a 100- to 300-km depth (Fig. 1) via hydrous carbonatitic melts (*20*–*23*). Reaction of these melts with overlying mantle may form carbonate-bearing peridotite (*24*) or carbonate-rich and clinopyroxene-rich (± amphibole, apatite, or phlogopite) metasomes (*6*, *18*, *25*), which, when undergoing subsequent melting, may produce carbonatitic liquids directly either via incipient melting (*26*) or via evolution/unmixing of carbonated silicate melt (*27*, *28*). For further discussion on the genesis and evolution of carbonatite magmas in the lithosphere, the reader is referred to review papers in refs. (*24*) and (*28*). A test of the subduction metasomatism model is whether occurrences of alkaline magmatism, including carbonatites, and their critical metal ores can be spatially and genetically linked to previous subduction-related mantle fertilization events.

There is an emerging consensus that plate subduction extends back to at least 3.1 billion years ago (Ga) (*29*). This onset broadly corresponds to the recorded age range of carbonatites and other intraplate alkaline magmatism (*2*, *30*). Advances in plate tectonic modeling now provides spatial reconstructions of plate kinematics, including convergent plate boundaries, back to 2 Ga (*31*–*33*). Here, we use these plate models to identify continental domains with FML and compare these domains to the location of carbonatites and alkaline magma–related REE ore deposits at present day. Our analysis not only serves to test a leading model for formation of intraplate alkaline magmatism back through Earth history but also provides a platform to reduce the global search space for new critical mineral ore discoveries.

Our approach uses full-plate reconstructions (*31*–*33*) constructed using GPlates (*34*) to identify plate convergence zones along continental margins from 2 Ga to the present day (see Materials and Methods). We define convergence zones with the greatest potential for mantle lithosphere metasomatism by using two criteria:

1) We only consider plate convergence episodes as lasting continuously for at least 100 million years (Myr). This time frame approximates the duration of long-lived continental arcs (*35*, *36*) that have demonstrable metasomatized mantle lithosphere [e.g., (*37*–*39*)] and is longer than oceanic arc and inter-arc magmatic events (e.g., flare-ups) that typical last <70 Myr (*40*). A 100-Myr duration is also appropriate for considering Precambrian plate convergence where current kinematic plate models have greatly reduced temporal resolution (*32*).

2) We only consider subduction systems where the location of the trench remains fixed relative to the adjacent continental block, thereby providing maximum metasomatic flux to the adjacent subcontinental mantle lithosphere. This does exclude long-lived accretionary convergent margins where substantial (>1000 km) trench migration has occurred (e.g., Phanerozoic eastern Australia).

We define polygons of FML domains adjacent to these long-lived convergent margins, extending along the length of the continental margin, and from the trench inboard for 900 km (Fig. 1). The polygons are then locked to the associated continental plate for subsequent tracking of these FML domains forward through time. The distance inboard of 900 km was constrained by considering:

1) Evidence from xenoliths and back-arc volcanic rocks of modern continental arcs [for example, from the southern Andes (*37*), western USA (*38*, *41*), eastern China (*39*), and eastern Aleutians (*42*)] that indicate subduction-related metasomatism of the mantle lithosphere extends 600 to >1000 km inboard of the plate margin.

2) Subducting slabs were likely shallow(er) dipping during the Proterozoic (*29*, *43*) when mantle geotherms were notably higher than the modern Earth (*43*, *44*), meaning that Proterozoic subduction-related mantle metasomatism may also have extended large distances [>600 km (*43*)] inboard of the plate margin.

Our approach does not consider or capture other processes (e.g., short-duration subduction, mantle plume-lithosphere interaction, mantle lithosphere, and lower crustal delamination and melting) that may also produce metasomatized mantle lithosphere [the Tethyan-Himalayan convergent system may be a good example; e.g., ref. (*16*)]. Instead, we focus on identifying the lithospheric domains (all else being equal) with the greatest potential for extensive metasomatism.

The identified FML domains are compared to locations of intraplate magmatism and ore formation through time using our global compilations of continental carbonatites (*n* = 304) and magma-related REE ore deposits (*n* = 108) (see Materials and Methods and table S1). We limit our analysis to test post-orogenic origins of carbonatites and ore deposits, so we only include occurrences that date from 1.8 Ga to present. Including older deposits is likely to introduce bias in the results toward events outside the time range available for existing plate models [i.e., 2.0 Ga to present day; (*31*–*33*)].

## RESULTS

### FML domains

**Fig. 2.**
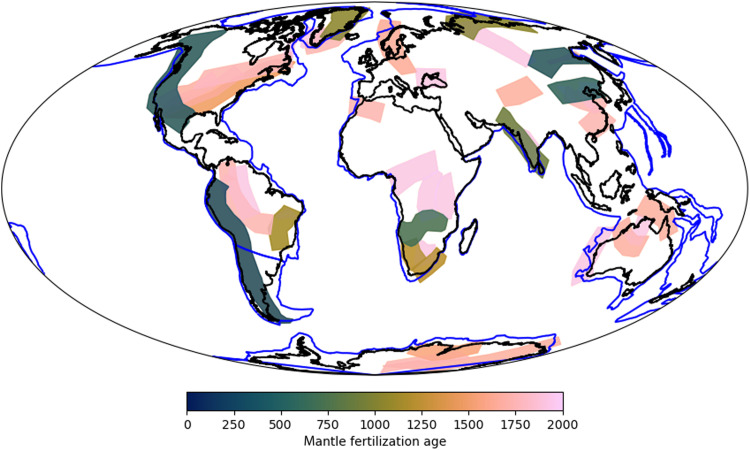
Present-day global distribution of FML polygons colored by age of polygon formation. Note that many polygons overlap with each other.

**Fig. 3.**
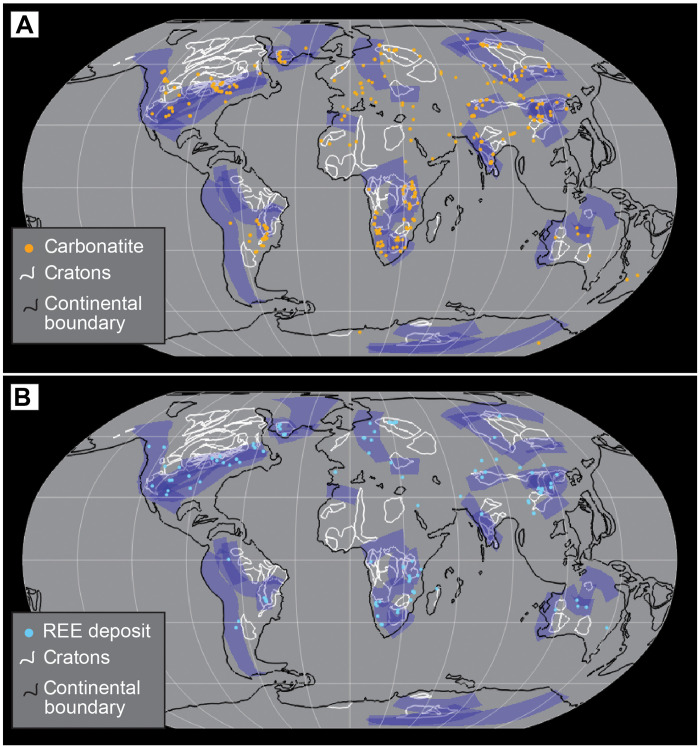
Present-day global distribution of carbonatites and magma-related REE ore deposits, together with FML polygons (in blue). (**A** and **B**) Carbonatites and REE ore deposits, respectively. Also shown are Archean cratons (white) and continental crust boundaries (black), adapted from (*58*).

**Fig. 4.**
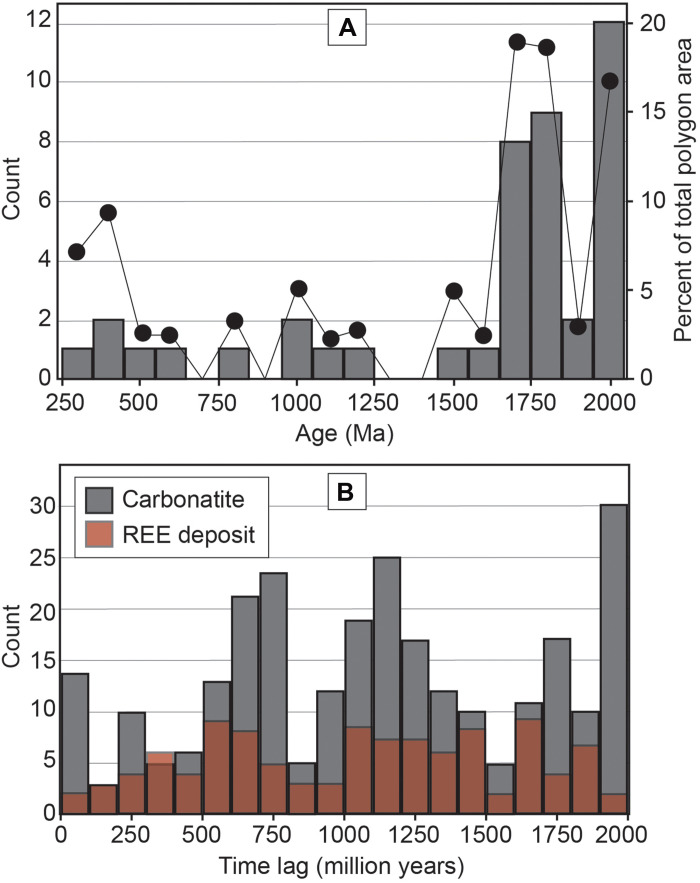
Histograms of FML polygon age and time lags. (**A**) Histogram of the count (gray columns, left axes) and relative area (black dots, right axis) of FML polygons as a function of their age. The polygon age is taken as the time of initiation of the subduction episode related to the polygon. (**B**) Histogram of the time lag (i.e., difference between the age of polygon and the age of carbonatite/REE ore deposit formation) for the carbonatites and REE deposits. Note that many carbonatites and REE ore deposits fall into more than one polygon.

We have mapped 43 polygons at present day, representing FML domains formed since 2.0 Ga. The duration of subduction fertilization for the polygons ranges from 100 to >500 Myr. The FML polygons are distributed across the globe but, in most cases (~85% of polygons), overlap other polygons due to repeated episodes of prolonged plate convergence (Fig. 2). Zones where multiple polygons overlap correspond with a greater proportion of carbonatites and REE deposits (for example, North America, southern Africa, and China; Fig. 3). The polygon ages range from 2.0 Ga to ~250 million years ago (Ma), although most (~75%) form between 2.0 and 1.7 Ga (Fig. 4A). There is a dearth of polygons aged between 1.5 and 0.7 Ga, which falls into Earth’s “middle ages,” a period of relatively subdued tectonic activity (*44*). The Phanerozoic polygons tend to be larger in area (table S2) due to the smaller area of recognized continental crust earlier in Earth history and due to splitting of some polygons during postformation continental rifting events. Most polygons (36 of 43) lie adjacent to, or overlapping, Archean cratonic blocks (Fig. 3). The total present-day identified area of our FML domains is ~75 million km^2^, which equates to 35% of the total area of Earth’s continental crust.

### Correlation to carbonatites and REE deposits

**Fig. 5.**
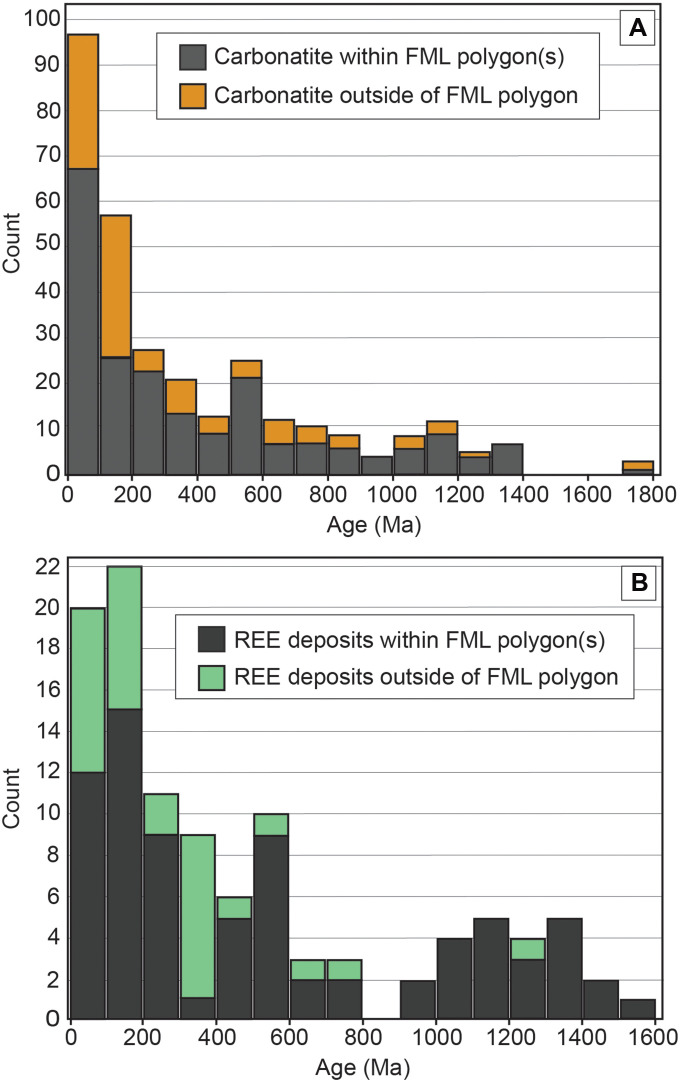
Histograms of the age of post–1.8-Ga carbonatites and REE ore deposits. (**A** and **B**) Carbonatites and REE ore deposits, respectively. Note the relatively young age distribution of the carbonatites and the high percentage (92%) of Precambrian REEs ore deposits contained within FML polygons.

Comparison of carbonatite and REE ore deposit localities to our FML polygons reveal that 67% (203 of 304) of carbonatites and 72% (78 of 108) of REE deposits fall within the FML domains (Fig. 3). FML polygons adjacent to Archean cratons tend to contain a larger number of carbonatites and REE deposits [Fig. 3 and fig. S1; see also (*30*)]. Expectedly, polygons in Antarctica include no identified carbonatites or REE deposits due to the almost complete ice cover. Many carbonatite and REE ore systems are known to form along major lithospheric scale structures (*10*, *45*, *46*), which may allow lateral transfer of these systems away from the source region. Therefore, we also consider “near miss” cases where carbonatites or deposits sit within 100 km of an FML polygon. For this case, our results reach 73 and 77% of carbonatites and REE deposits, respectively. Because many of the world’s largest and highest-grade REE ore deposits formed in the Proterozoic (*10*, *11*, *45*), we also analyzed only Precambrian deposits and found that 92% (34 of 37) of REE deposits (and 72% of carbonatites) lie within FML domains (Fig. 5).

For the carbonatites and REE deposits that do not fall within our FML polygons, we expect that many of them may have been emplaced in pre–2.0-Ga FML domains that our model does not capture. In support of this assumption, we note that a high proportion of carbonatites sit within early Proterozoic to Archean terranes (*30*, *47*).

To assess the veracity of our results, we conducted a random assignment test, where a healpix mesh was used to assign control points across the globe, resulting in a subset of 5074 points within continental crust. We then randomly selected 304 points (matching the number of carbonatites in our dataset) from the subset to see how many would fall within the FLM polygons and repeated the exercise 2000 times. The mean count of points within the FML polygons is 101 of 304 points, or 33%, which, expectedly, is similar to the value of 35% of the total continental crust covered by FML.

The time lag between polygon formation and formation of the carbonatite or REE deposit (Fig. 4B) varies widely and does not show systematic trends, other than a high proportion of the youngest carbonatites (i.e., <100 Ma) being associated with the oldest (~2.0 Ga) polygons. This may simply be reflecting the high number of Paleoproterozoic polygons (Fig. 4A) and young carbonatites (Fig. 5) or may be due to preservation bias, where many older deposits have already been eroded. The otherwise wide distribution of the time lags reflects the absence of direct connection between the processes forming the FML (i.e., long-lived subduction) and those forming carbonatites and REE deposits.

## DISCUSSION

Mantle lithosphere metasomatized by subducted-related fluids or melts has long been proposed as a source for continental intraplate magmatism [e.g., (*4*, *15*, *48*)]. Although many specific intraplate magmatic occurrences have been linked to past subduction events, we present a global-scale spatiotemporal analysis linking plate convergence episodes and alkaline intraplate magmatism back to the Paleoproterozoic. The significant correlation between the location of carbonatites and REE deposits with our FML domains provides a compelling case that the subduction-fertilized mantle lithosphere represents a viable source for continental alkaline magmatism (*9*) and associated critical mineral ore systems (*10*, *16*, *45*). This premise is also consistent with the general case that continental carbonatites and associated ore deposits have more enriched radiogenic isotope signatures than oceanic carbonatites and other mantle-derived magmas (*6*, *16*) and that carbonatites have stable isotope compositions (e.g., C, Mg, and B) that are broadly comparable with components of subducted lithosphere (*6*, *49*–*51*). Subduction along stable cratons may form especially fertile domains (Fig. 3) as, in these cases, subduction dynamics can be stabilized for long periods, maximizing the potential for mantle lithosphere metasomatism.

Our results suggest that processes such as upwelling mantle plumes (*7*, *12*, *13*) or fluxes from deep-mantle slab graveyards (*8*) are not required to produce the source regions for alkaline intraplate magmatism, although it also does not exclude these mechanisms. We do note, however, the lack of spatial correlation between carbonatites and REE deposits and large igneous provinces that are widely regarded to be magmatic products of mantle plumes (*52*) (fig. S2).

The variable and sometimes extensive time lag between stages of fertilization and subsequent alkaline magmatism (Fig. 4B) is consistent with the observed temporal disconnect between plate boundary processes and intraplate magmatism: It is consistent with observations of (*53*) that carbonatites can form repeatedly in similar locations but at very different time periods. On this basis, we propose that intraplate alkaline magmatism, in many cases, develops via two temporally disconnected stages: an initial stage of the mantle source metasomatism, which we consider the “primer” to magmatism, and a subsequent melting “trigger” stage responsible for the generation of the magma. Moreover, this time lag [up to 2 billion years (Gyr)] indicates that FML, once produced, can be preserved for long periods. At first, this appears inconsistent with the general view that the enriched mantle lithosphere is relatively dense and susceptible to destruction by asthenospheric convection. However, mantle metasomatism by subduction-zone recycling is likely to produce hydrous (amphibole and phlogopite) and carbonate rich domains [or metasomes; (*4*, *5*, *18*)] within the lithosphere, which are expected to be of relatively low density and hence gravitationally stable. This is also consistent with demonstrated long-term stability of the cratonic lithosphere as recently reported in ref. (*54*). In this case, FML domains may also represent major long-term stores of H_2_O and carbon (*55*).

Our analysis is based on correlation between present-day deposits and areas of past mantle fertilization; we do not model the causative mechanism of partial melting and generation of the alkaline magma (i.e., the “trigger”). Zones of FML are expected to have much lower solidi relative to the ambient upper mantle (*4*, *5*, *9*), which means they would be susceptible to low-degree partial melting (the conditions needed for generation of highly alkaline magma), even by mild thermal or structural perturbations of the lithosphere. The large range of recorded lag times between source enrichment and melting (Fig. 4B) suggests that a range of the potential processes may be triggers for magma production (*9*). This may include deformation of the lithosphere due to an impinging mantle plume or intracontinental extension (*12*, *52*), asthenospheric flow around a lithospheric step (*9*), or even mantle decompression due to deglaciation (*56*). Considering this latter case, it is noteworthy that five FML polygons underlie glaciated regions of Antarctica and Greenland. Deglaciation (for example, under a warming global climate) may therefore be a trigger for the initiation of intraplate volcanism in these regions in the future.

A high proportion of carbonatites and REE deposits is Phanerozoic in age (*2*, *30*, *45*, *47*) (Fig. 5). Although these statistics may in part reflect a preservation bias and the fact that a large fraction of exposed continental crust is also of Phanerozoic age, they may also reflect the greater proportion of fertile source regions for carbonatites and REE deposits in recent times compared to the Precambrian, simply due to the progressive addition of FML domains since 2.0 Ga (and assuming a high preservation rate of FML, as discussed above). The diverse radiogenic isotope composition of Phanerozoic carbonatites (*6*, *16*) is also consistent with this premise. Secular cooling of the Earth may also play a role as cooler upper mantle conditions are arguable more optimal for producing fertile low-degree mantle melts, and hence a diverse range of processes [mantle plumes, lithospheric steps, continental rifting, and lithospheric disruption due to far-field effects (*9*)] may be able to produce alkaline magmatism and associated ore systems in the modern Earth.

Our model also has important implications for regional prospectivity for economic deposits of REE and other critical minerals. Recognition that FML domains host a majority of REE deposits, including ~92% of Precambrian deposits, means that these zones can be used as a first-order target for mineral exploration. More specific targeting may benefit from spatial correlation between FML domains, cratons, and lithospheric structures or steps (*9*, *10*, *53*), which are also recognized to control the location of REE deposits (*10*, *16*, *45*, *46*). Identifying magmas formed via low-degree mantle melting, a prerequisite for REE ore formation (*10*, *45*), would also assist efforts to locate previously unrecognized orebodies. We expect that future refinements to paleotectonic models, including further back through time, will enhance these models and may also be used to identify other ore systems linked to the enriched mantle lithosphere, such as orthomagmatic Ni deposits (*27*, *57*), many of which are Archean to Paleoproterozoic in age.

## MATERIALS AND METHODS

Mapping of FML polygons was conducted using the GPlates software (*34*), using plate kinematic models and continental configurations of (*32*) and (*33*) for tectonic models from 1.8 Ga to present day and (*31*) for 1.8 to 2.0 Ga. The model of (*32*) is our preferred model as it is based on a wide range of available geological and geophysical constraints but only extends to 1.8 Ga. Therefore, the model of (*31*) is used to extend our analysis from 1.8 to 2.0 Ga. FML polygons were identified by tracking convergent margins adjacent to continental blocks through time. Only convergent margins operating for more than 100 Myr and with a fixed trench location along the continental margin were selected for FML polygon assignment.

Polygons were manually drawn (in GPlates) overlying the margin and continental plate from the trench inboard for 900 km and along the length of the convergent margin. The polygon was then fixed to the continental plate to allow tracking forward through time. If the continental plate undergoes rifting to form two separate plates at a later (younger) time, then the polygon was also redrawn as two abutting polygons to allow each to separate with its associated rifted plate segment. The polygon data were recorded as a series shapefiles defined by time periods of Phanerozoic, Neoproterozoic, and Mesoproterozoic, the latter including one shapefile from 1.8 to 2.0 Ga [i.e., based on (*31*)] and one from 1.0 to 1.8 Ga using the reconstruction of (*32*). At present day, many of the polygons overlap each other (Fig. 2); the calculated total present-day polygon area accounts for these overlaps.

Plate reconstruction models in the Proterozoic are acknowledged to be less reliable and of lower resolution that Phanerozoic models [e.g., (*33*)]. Nevertheless, we are confident of the veracity of our FML modeling as a key model parameter is to only include long-lived (>100 Myr) subduction systems; for these, there is substantive supporting evidence from the geological record (*58*).

Compilations of carbonatites and REE ore deposits only include those in continental crust and those formed after 1.8 Ga. Details of the source data for these compilations are provided in table S1. Our compilation only includes carbonatite occurrences that conform with recent carbonatite classification criteria [e.g., (*59*)], have robust age constraints, and are of substantial documented extent (i.e., more than a few thin veins). The REE ore deposit dataset extends the compilation of (*45*) and only includes deposits for which there are clear origins from intraplate alkaline magmatism. We exclude deposits lacking robust age constraints and where mineralization solely relates to supergene or weathering processes. Nonetheless, our deposit list still covers most global REE reserves [e.g., (*10*, *11*, *45*)]. Most of these deposits not only are carbonatite related but also include those related alkaline silicate intrusions and volcanic complexes. The locations and age of the carbonatites and REE ore deposits were captured as shapefiles to allow import into GPlates, where our spatiotemporal analysis and association with the FML polygons and other geological features [for example, cratons and large igneous provinces from (*58*)] were completed. The Python code used to facilitate our analysis can be found at data S1.

## Supplementary Materials

### The PDF file includes:

Figs. S1 and S2 Legends for tables S1 and S2 Legend for data S1 References
