# Supplementary material for: Linking carbonatites, rare earth ores, and subduction-fertilized mantle lithosphere
Source: Sci Adv. 2026 Apr 8;12(15):eaeb2942. doi: 10.1126/sciadv.aeb2942 (PMC13060588; doi:10.1126/sciadv.aeb2942)
Supplement: Supplementary file 1 — Figs. S1 and S2 Legends for tables S1 and S2 Legend for data S1 References [file sciadv.aeb2942_sm.pdf]

Science Advances
AAAS

Supplementary Materials for

Linking carbonatites, rare earth ores, and subduction-fertilized mantle lithosphere

Carl Spandler *et al.*

Corresponding author: Carl Spandler, carl.spandler@adelaide.edu.au

Sci. Adv. **12**, eaeb2942 (2026)
DOI: 10.1126/sciadv.aeb2942

The PDF file includes:

Figs. S1 and S2
Legends for tables S1 and S2
Legend for data S1
References

Other Supplementary Material for this manuscript includes the following:

Tables S1 and S2
Data S1

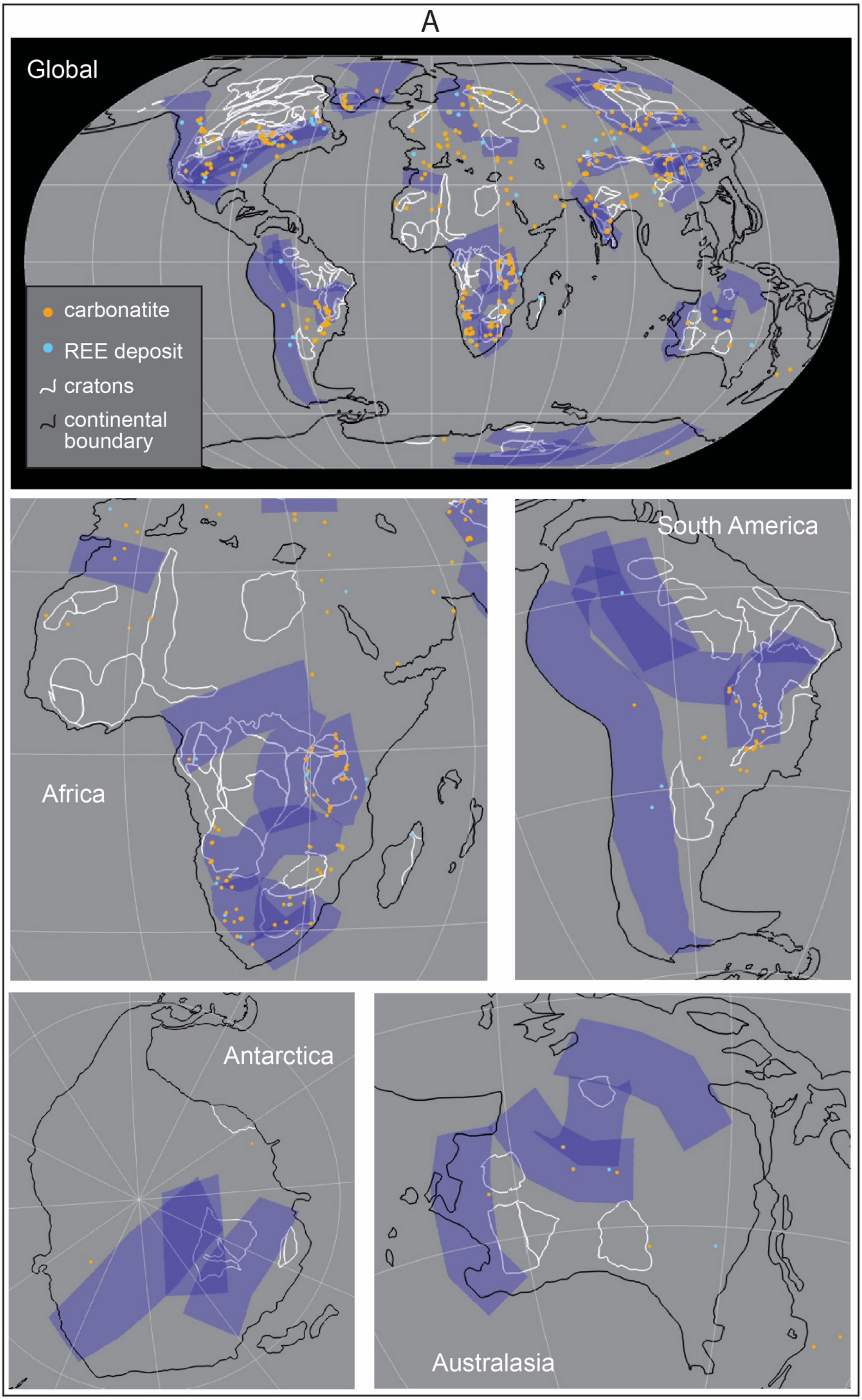
A
Global
carbonatite
REE deposit
cratons
continental boundary
Africa
South America
Antarctica
Australasia

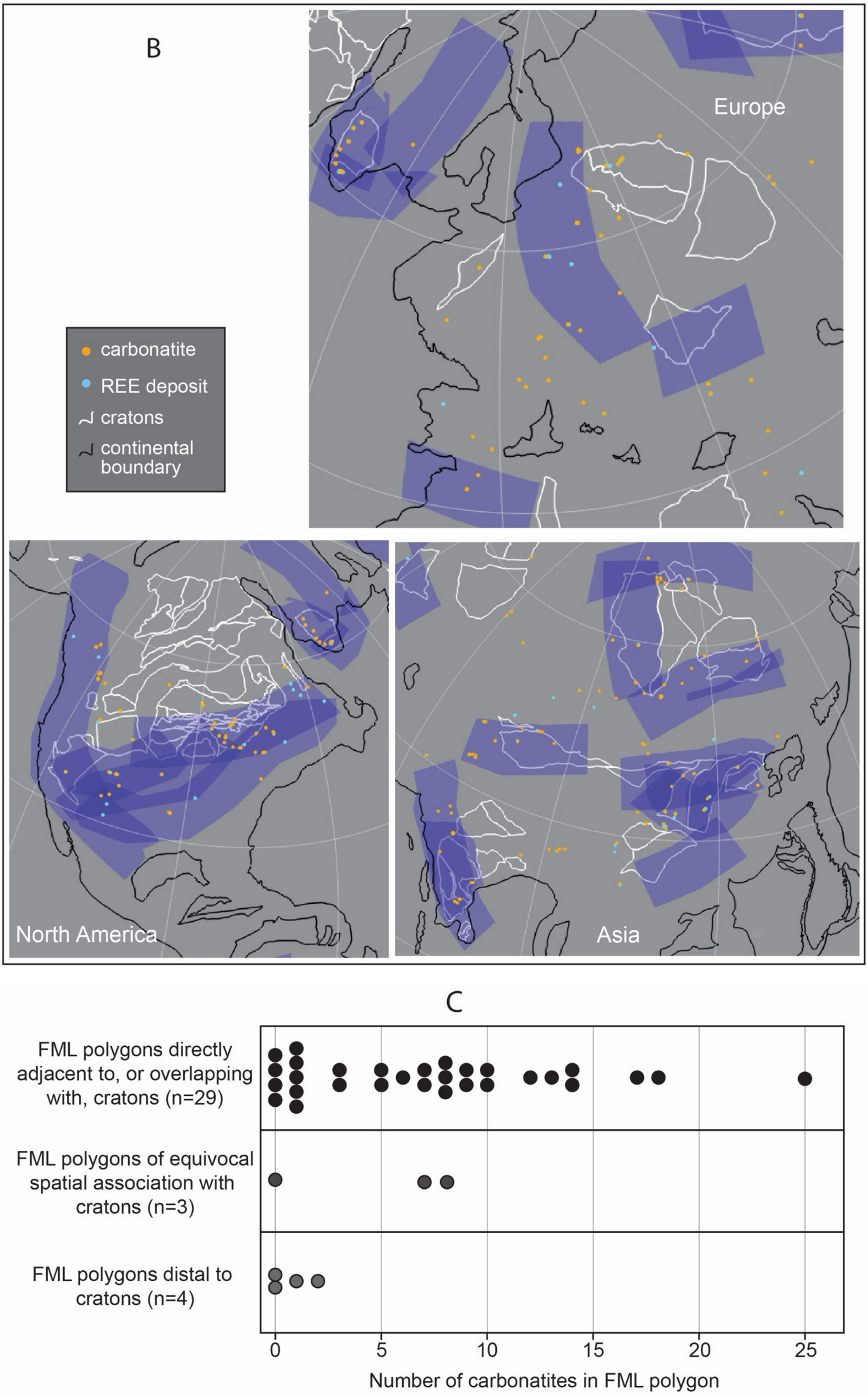
B
Europe
carbonatite
REE deposit
cratons
continental boundary
North America
Asia
C
FML polygons directly adjacent to, or overlapping with, cratons (n=29)
FML polygons of equivocal spatial association with cratons (n=3)
FML polygons distal to cratons (n=4)
0
5
10
15
20
25
Number of carbonatites in FML polygon

Supplementary Figure S1. Present-day distribution of carbonatites, magma-related REE ore deposits, FML polygons, Archean cratons and continental crust. (**A)** Present-day global and southern hemisphere distribution of carbonatites, magma-related REE ore deposits, FML polygons (in blue), Archean cratons (white) and continental crust boundaries (black). The craton and continental boundaries are adapted from ref (*58*). **(B)** Present-day northern hemisphere distribution of carbonatites, magma-related REE ore deposits, FML polygons (in blue), Archean cratons (white) and continental crust boundaries (black). The craton and continental boundaries are adapted from ref (*58*). Note the association of carbonatites and REE ore deposits with FML polygons directly adjacent to, or overlapping, cratons. **(C)** Qualitative analysis of FML polygon relationship with Archean cratonic blocks and the number of carbonatites within these polygons. Note, this analysis excludes polygons from Antarctica as there are no carbonatites recognised from within these polygons due to almost complete ice cover.

Supplementary Figure S2. Present-day global maps of large igneous provinces compared to the location of carbonatites and REE ore deposits. Panels **(A)** and **(B)** present carbonatites and REE ore deposits, respectively. Overall, there is no strong correlation between the large igneous provinces and carbonatites and REE ore deposits. The location of the large igneous provinces is taken from ref *(58)*.

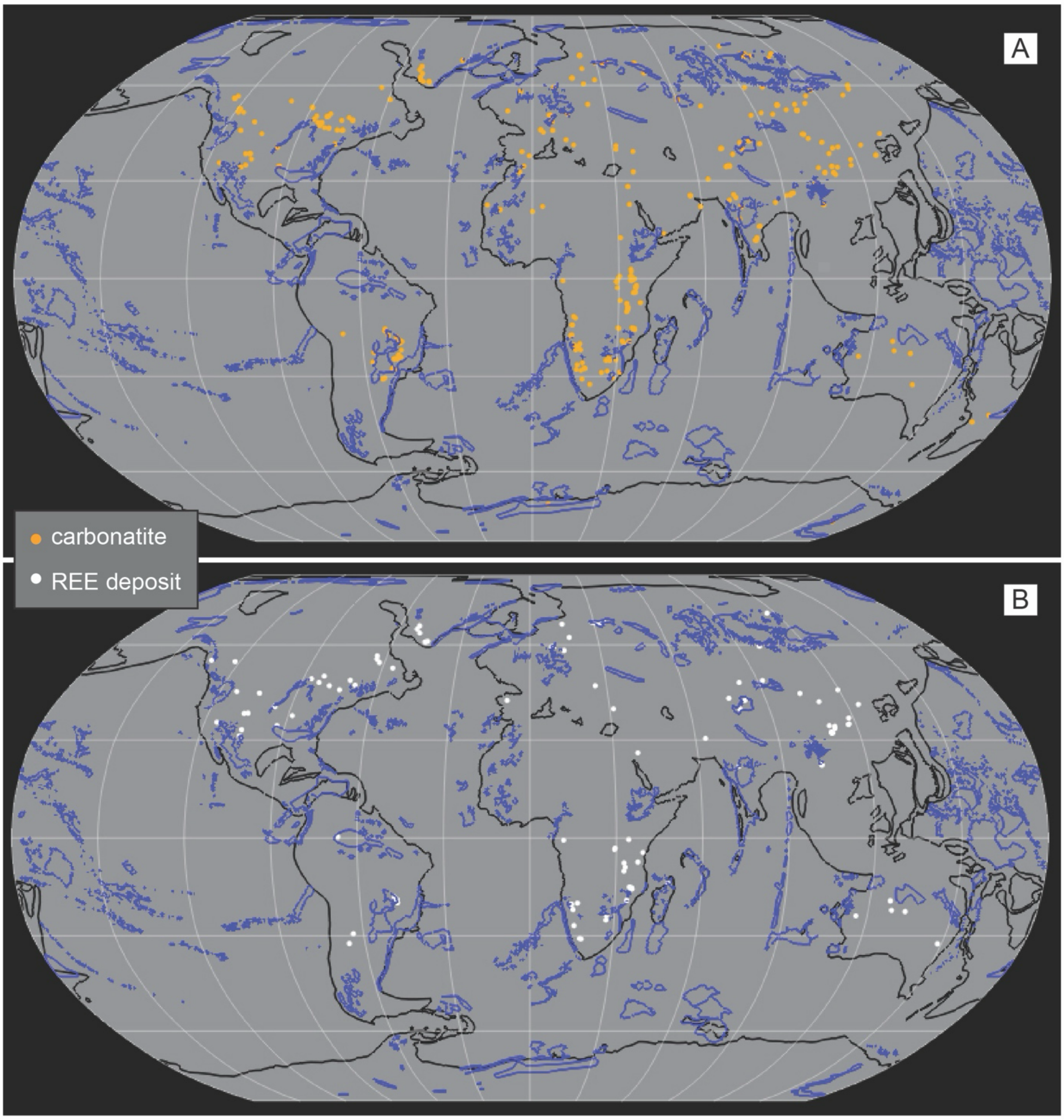

Supplementary Table Captions

Supplementary Table S1. Carbonatite and REE ore deposit datasets, and model results. **(A)** Carbonatite dataset. Age and location data from (*2,11,28*). **(B)** Magma-related REE ore deposit dataset. Age and location data from (*10,11,16,28,45,60-68*). **(C)** Model results.

Supplementary Table S2. Details of the age, size and location of FML polygons and their contained carbonatite and REE ore deposits.

Data S1. Supplementary datasets.

1. shapefiles of FML polygons, carbonatite and REE ore deposit locations, and continent outlines
2. Data tables identifying carbonatites and REE ore deposits that lie within FML polygons

REFERENCES

1. G. L. Farmer, "Continental basaltic rocks" in *Treatise on Geochemistry, Volume 4: The Crust*, H. D. Holland, K. K. Turekian, Eds. (Elsevier, 2003), pp. 85–121.

2. S. L. Liu, L. Ma, X. Zou, L. Fang, B. Qin, A. E. Melnik, U. Kirscher, K.-F. Yang, H.-R. Fan, R. N. Mitchell, Trends and rhythms in carbonatites and kimberlites reflect thermo-tectonic evolution of Earth. *Geology* **51**, 101–105 (2023).

3. K. D. Litasov, A. Shatskiy, E. Ohtani, G. M. Yaxley, Solidus of alkaline carbonatite in the deep mantle. *Geology* **41**, 79–82 (2013).

4. S. Pilet, M. B. Baker, E. M. Stolper, Metasomatized lithosphere and the origin of alkaline lavas. *Science* **320**, 916–919 (2008).

5. S. F. Foley, I. S. Ezad, S. R. van der Laan, M. Pertermann, Melting of hydrous pyroxenites with alkali amphiboles in the continental mantle: 1. Melting relations and major element compositions of melts. *Geosci. Front.* **13**, 101380 (2022).

6. G. M. Yaxley, M. Anenburg, S. Tappe, S. Decree, T. Guzmics, Carbonatites: Classification, sources, evolution, and emplacement. *Annu. Rev. Earth Planet. Sci.* **50**, 261–293 (2022).

7. R. E. Ernst, K. L. Buchan, Recognizing mantle plumes in the geological record. *Annu. Rev. Earth Planet. Sci.* **31**, 469–523 (2003).

8. B. R. Mather, R. D. Müller, M. Seton, S. Ruttor, O. Nebel, N. Mortimer, Intraplate volcanism triggered by bursts in slab flux. *Sci. Adv.* **6**, eabd0953 (2020).

9. S. Gibson, D. McKenzie, S. Lebedev, The distribution and generation of carbonatites. *Geology* **52**, 667–671 (2024).

10. C. D. Beard, K. M. Goodenough, A. M. Borst, F. Wall, P. R. Siegfried, E. A Deady, C. Pohl, W. Hutchison, A. A. Finch, B. F. Walter, H. A. L. Elliott, K. Brauch, Alkaline-silicate REE-HFSE systems. *Econ. Geol.* **118**, 177–208 (2023).

11. M. P. Smith, K. Moore, D. Kavecsánszki, A. A. Finch, J. Kynicky, F. Wall, From mantle to critical zone: A review of large and giant sized deposits of the rare earth elements. *Geosci. Front.* **7**, 315–334 (2016).

12. F. Pirajno, Intracontinental anorogenic alkaline magmatism and carbonatites, associated mineral systems and the mantle plume connection. *Gondwana Res.* **27**, 1181–1216 (2015).

13. K. Bell, "Carbonatites: Relationships to mantle-plume activity" in *Mantle Plumes: Their Identification Through Time*, R. E. Ernst, K. L. Buchan, Eds. (Geological Society of America, 2001), vol. **352**.

14. W. Dan, Q. Wang, G. J. Tang, X. Z. Zhang, Magmatism in continental rifts and rifted margins. *Sci. China Earth Sci.* **68**, 3986–4004 (2025).

15. Z.-Q. Hou, B. Xu, H. Zhang, Y.-C. Zheng, R. Wang, Y. Liu, Z. Miao, L. Gao, Z. Zhao, W. L. Griffin, S. Y. O'Reilly, Refertilized continental root controls the formation of the Mianning–Dechang carbonatite-associated rare-earth-element ore system. *Commun. Earth Environ.* **4**, 293 (2023).

16. K. M. Goodenough, E. A. Deady, C. D. Beard, S. Broom-Fendley, H. A. Elliott, F. van den Berg, H. Öztürk, Carbonatites and alkaline igneous rocks in post-collisional settings: Storehouses of rare Earth elements. *J. Earth Sci.* **32**, 1332–1358 (2021).

17. C. Spandler, C. Pirard, Element recycling from subducting slabs to arc crust: A review. *Lithos* **170–171**, 208–223 (2013).

18. F. Gülmez, D. Prelević, M. W. Förster, S. Buhre, J. Günther, Experimental production of K-rich metasomes through sediment recycling at the slab-mantle interface in the fore-arc. *Sci. Rep.* **13**, 19608 (2023).

19. D. Prelević, M. W. Förster, S. Buhre, F. Gülmez, T. Grützner, Y. Wang, S. F. Foley, Recent advances made by reaction experiments on melting of heavily metasomatized hydrous mantle. *Earth Sci. Rev.* **256**, 104881 (2024).

20. S. Poli, Carbon mobilized at shallow depths in subduction zones by carbonatitic liquids. *Nat. Geosci.* **8**, 633–636 (2015).

21. L. A. Martin, J. Hermann, Experimental phase relations in altered oceanic crust: Implications for carbon recycling at subduction zones. *J. Petrol.* **59**, 299–320 (2018).

22. Y. Zhang, C. Wang, W. Li, Z. Jin, Carbon dioxide released from subducted oceanic crust by hydrous carbonatitic liquids. *Geophys. Res. Lett.* **50**, e2023GL104734 (2023).

23. W. Chen, G. Zhang, S. Keshav, Y. Li, Pervasive hydrous carbonatitic liquids mediate transfer of carbon from the slab to the subarc mantle. *Commun. Earth Environ.* **4**, 73 (2023).

24. G. M. Yaxley, S. Ghosh, E. S. Kiseeva, A. Mallik, C. Spandler, A. R. Thomson, M. J. Walter, "CO_2-rich melts in Earth" in *Deep Carbon: Past to Present*, B. N. Orcutt, I. Daniel, R. Dasgupta, Eds. (Cambridge Univ. Press, 2019), pp. 129–162.

25. X. Wang, J. Zhang, C. Wang, K. Zong, H. Xu, Experimental constraint on Ca-rich carbonatite melt-peridotite interaction and implications for lithospheric mantle modification beneath the North China craton. *J. Geophys. Res. Solid Earth* **127**, e2022JB024769 (2022).

26. I. S. Ezad, M. Saunders, S. S. Shcheka, M. L. Fiorentini, L. R. Gorojovsky, M. W. Förster, S. F. Foley, Incipient carbonate melting drives metal and sulfur mobilization in the mantle. *Sci. Adv.* **10**, eadk5979 (2024).

27. C. Chen, M. W. Förster, S. S. Shcheka, I. S. Ezad, J. J. Shea, Y. Liu, D. E. Jacob, S. F. Foley, Sulfide-rich continental roots at cratonic margins formed by carbonated melts. *Nature* **637**, 615–621 (2025).

28. M. W. Schmidt, A. Giuliani, S. Poli, The origin of carbonatites—Combining the rock record with available experimental constraints. *J. Petrol.* **65**, egae105 (2024).

29. R. M. Palin, M. Santosh, Plate tectonics: What, where, why, and when? *Gondwana Res.* **100**, 3–24 (2021).

30. E. R. Humphreys-Williams, S. Zahirovic, Carbonatites and global tectonics. *Elements* **17**, 339–344 (2021).

31. Z. X. Li, Y. Liu, R. Ernst, A dynamic 2000—540 Ma Earth history: From cratonic amalgamation to the age of supercontinent cycle. *Earth Sci. Rev.* **238**, 104336 (2023).

32. X. Cao, A. S. Collins, S. Pisarevsky, N. Flament, S. Li, D. Hasterok, R. D. Müller, Earth's tectonic and plate boundary evolution over 1.8 billion years. *Geosci. Front.* **15**, 101922 (2024).

33. A. S. Merdith, S. E. Williams, A. S. Collins, M. G. Tetley, J. A. Mulder, M. L. Blades, A. Young, S. E. Armistead, J. Cannon, S. Zahirovic, R. D. Müller, Extending full-plate tectonic models into deep time: Linking the Neoproterozoic and the Phanerozoic. *Earth Sci. Rev.* **214**, 103477 (2021).

34. R. D. Müller, J. Cannon, X. Qin, R. J. Watson, M. Gurnis, S. Williams, T. Pfaffelmoser, M. Seton, S. H. J. Russell, S. Zahirovic, GPlates: Building a virtual Earth through deep time. *Geochem. Geophys. Geosyst.* **19**, 2243–2261 (2018).

35. W. Cao, C. T. A. Lee, J. S. Lackey, Episodic nature of continental arc activity since 750 Ma: A global compilation. *Earth Planet. Sci. Lett.* **461**, 85–95 (2017).

36. M. N. Ducea, J. B. Saleeby, G. Bergantz, The architecture, chemistry, and evolution of continental magmatic arcs. *Annu. Rev. Earth Planet. Sci.* **43**, 299–331 (2015).

37. G. Rivalenti, M. Mazzucchelli, A. Laurora, S. I. Ciuffi, A. Zanetti, R. Vannucci, C. A. Cingolani, The backarc mantle lithosphere in Patagonia, South America. *J. South Am. Earth Sci.* **17**, 121–152 (2004).

38. G. L. Farmer, D. E. Fritz, A. F. Glazner, Identifying metasomatized continental lithospheric mantle involvement in Cenozoic magmatism from Ta/Th values, southwestern North America. *Geochem. Geophys. Geosyst.* **21**, e2019GC008499 (2020).

39. M. Sun, H. Chen, L. A. Milan, S. A. Wilde, F. Jourdan, Y. Xu, Continental arc and back-arc migration in Eastern NE China: New constraints on Cretaceous Paleo-Pacific subduction and rollback. *Tectonics* **37**, 3893–3915 (2018).

40. S. R. Paterson, M. N. Ducea, Arc magmatic tempos: Gathering the evidence. *Elements* **11**, 91–98 (2015).

41. K. Putirka, B. Platt, Basin and Range volcanism as a passive response to extensional tectonics. *Geosphere* **8**, 1274–1285 (2012).

42. A. Tibaldi, F. L. Bonali, Intra-arc and back-arc volcano-tectonics: Magma pathways at Holocene Alaska-Aleutian volcanoes. *Earth Sci. Rev.* **167**, 1–26 (2017).

43. A. L. Perchuk, V. S. Zakharov, T. V. Gerya, R. J. Stern, Shallow vs. Deep subduction in Earth history: Contrasting regimes of water recycling into the mantle. *Precambrian Res.* **418**, 107690 (2025).

44. P. A. Cawood, P. Chowdhury, J. A. Mulder, C. J. Hawkesworth, F. A. Capitanio, P. M. Gunawardana, O. Nebel, Secular evolution of continents and the Earth system. *Rev. Geophys.* **60**, e2022RG000789 (2022).

45. C. Spandler, P. Slezak, T. Nazari-Dehkordi, Tectonic significance of Australian rare earth element deposits. *Earth Sci. Rev.* **207**, 103219 (2020).

46. Y. Xie, Z. Hou, R. J. Goldfarb, X. Guo, L. Wang, Rare earth element deposits in China. *Rev. Econ. Geol.* **18**, 115–136 (2016).

47. A. R. Woolley, B. A. Kjarsgaard, *Carbonatite Occurrences of the World: Map And Database* (Geological Survey of Canada, 2008), 28 pp.

48. C. J. Hawkesworth, N. W. Rogers, P. W. C. Van Calsteren, M. A. Menzies, Mantle enrichment processes. *Nature* **311**, 331–335 (1984).

49. J. S. Ray, R. Ramesh, Stable carbon and oxygen isotopic compositions of Indian carbonatites. *Int. Geol. Rev.* **48**, 17–45 (2006).

50. T. Plank, C. E. Manning, Subducting carbon. *Nature* **574**, 343–352 (2019).

51. S. R. Hulett, A. Simonetti, E. T. Rasbury, N. G. Hemming, Recycling of subducted crustal components into carbonatite melts revealed by boron isotopes. *Nat. Geosci.* **9**, 904–908 (2016).

52. R. E. Ernst, K. Bell, Large igneous provinces (LIPs) and carbonatites. *Mineral. Petrol.* **98**, 55–76 (2010).

53. A. R. Woolley, D. K Bailey, The crucial role of lithospheric structure in the generation and release of carbonatites: Geological evidence. *Mineral. Mag.* **76**, 259–270 (2012).

54. Z. J. Sudholz, K. Priestley, A. Copley, Long-term evolution, stability, and thickness of cratonic lithosphere. *Geology* **53**, 1012–1016 (2025).

55. S. F. Foley, T. P. Fischer, An essential role for continental rifts and lithosphere in the deep carbon cycle. *Nat. Geosci.* **10**, 897–902 (2017).

56. D. E. Eason, J. M. Sinton, K. Grönvold, M. D. Kurz, Effects of deglaciation on the petrology and eruptive history of the Western Volcanic Zone, Iceland. *Bull. Volcanol.* **77**, 1–27 (2015).

57. I. S. Ezad, D. E. Blanks, S. F. Foley, D. A. Holwell, J. Bennett, M. L. Fiorentini, Lithospheric hydrous pyroxenites control localisation and Ni endowment of magmatic sulfide deposits. *Miner. Depos.* **59**, 227–236 (2024).

58. D. Hasterok, J. A. Halpin, A. S. Collins, M. Hand, C. Kreemer, M. G. Gard, S. Glorie, New maps of global geological provinces and tectonic plates. *Earth Sci. Rev.* **231** 104069 (2022).

59. R. H. Mitchell, Carbonatites and carbonatites and carbonatites. *Can. Mineral.* **43**, 2049–2068 (2005).

60. A. G. Marlow, M. R. Palmer, A preliminary study of the rare earth element-enriched Twyfelskupje carbonatite complex, southern Namibia. *Geol. Mag.* **160**, 305–321 (2023).

61. S. Yan, Q. Shan, H. C. Niu, X. Yu, X. Zhao, X. C. Zhao, H. J. Zhang, Y. Xiong, Timing and genesis of the Tudiling trachyte Nb-Ta-Zr-REE deposit in the South Qinling (Central China): Implications for rare metal enrichment in extrusive peralkaline magmatic systems. *Ore Geol. Rev.* **139**, 104535 (2021).

62. M. C. Moshi, Y. Watanabe, N. Boniface, T. Tsujimori, C. Tupaz, D. Araoka, S. Aoki, E. E. Mshiu, Petrological, geochemical and mineralogical characteristics of Wigu Hill carbonatite, Uluguru Mountains, Tanzania: Insights into carbonatite evolution and REE mineralization. *Miner. Depos.* **59**, 1755–1775 (2024).

63. W. Zhang, W. T. Chen, J. F. Gao, H. K. Chen, J. H. Li, Two episodes of REE mineralization in the Qinling Orogenic Belt, Central China: In-situ U-Th-Pb dating of bastnäsite and monazite. *Miner. Depos.* **54**, 1265–1280 (2019).

64. G. W. Huang, C. R. Pan, J. Y. Pan, F. J. Zhong, Z. L. Chen, F. Xia, J. Yan, D.-H. Wu, Z. Min, C. Bonnetti, J.-J. Wan, Q.-Q. Kang, REE mineralization age and geodynamic setting of the Huanglongpu deposit in the East Qinling orogen, China: Evidence from mineralogy, U–Pb geochronology, and in-situ Nd isotopes. *Ore Geol. Rev.* **152**, 105255 (2023).

65. M. Xie, W. Xiao, B. Su, P. A. Sakyi, S. Ao, J. Zhang, D. Song, Z. Zhang, Z. Li, C. Han, REE mineralization related to carbonatites and alkaline magmatism in the northern Tarim basin, NW China: Implications for a possible Permian large igneous province. *Int. J. Earth Sci.* **111**, 2759–2776 (2022).

66. E. V. Levashova, S. G. Skublov, T. A. Oitseva, B. A. Dyachkov, X.-H. Li, Q.-L. Li, N. V. Shatova, V. V. Shatov, First age and geochemical data on zircon from Riebeckite Granites of the Verkhnee Espe rare earth–rare metal deposit, East Kazakhstan. *Geochem. Int.* **60**, 1–15 (2022).

67. J.-H. Su, X.-F. Zhao, X.-C. Li, S.-R. Chang, Y.-B. Wu, C. Spandler, A linkage between early Silurian Nb-REE enriched alkaline magmatism and Neoproterozoic subduction metasomatized mantle in South Qinling, Central China. *Lithos* **440–441**, 107046 (2023).

68. C. Wang, J. F. Slack, A. K. Shah, M. G. Yates, D. R. Lentz, A. T. Whittaker, R. G. Marvinney, A recently discovered trachyte-hosted rare earth element-niobium-zirconium occurrence in northern Maine, USA. *Econ. Geol.* **118**, 1–13 (2023).
